# Silencing of circ_002136 sensitizes gastric cancer to paclitaxel by targeting the miR-16-5p/HMGA1 axis

**DOI:** 10.1515/med-2022-0625

**Published:** 2023-01-31

**Authors:** Zhijian Kuang, Haitao Yang, Shu Cheng, Xiaolong Zhou, Lan Chen, Yuqing Zhang, Jie Zhang

**Affiliations:** Department of Pathology, Ningbo Mingzhou Hospital, Ningbo, Zhejiang, China; Department of Pathology, The First People’s Hospital of Wenling, Wenling, Zhejiang, No. 333, Chuan’an South Road, Chengxi Street, Wenling, Zhejiang Province, 3175000, China

**Keywords:** gastric cancer, circ_002136, miR-16-5p, HMGA1, paclitaxel

## Abstract

The dysregulated expression of circRNA in gastric cancer (GC) induces paclitaxel (Tax) resistance of cancer cells, which in turn affects disease progression and prognosis. Here, we sought to investigate the role and mechanism of circ_002136 in Tax-resistant GC. In this study, we found the enriched circ_002136 level and the declined miR-16-5p level in Tax-resistant GC tissues and cells. Biologically, knockdown of circ_002136 elevated the Tax sensitivity of Tax-resistant GC cells, inhibited the cell motility properties, and simultaneously drove the apoptosis. Mechanically, circ_002136 promoted the HMGA1-mediated cellular Tax resistance and cell invasion by sponging miR-16-5p. Furthermore, circ_002136 silencing impeded the growth of Tax-resistant GC tumors *in vivo*. Overall, our study revealed a novel signaling pathway that could be used for future clinical applications, namely the circ_002136/miR-16-5p/HMGA1 axis to regulate the Tax resistance of GC cells.

## 1 Introduction

Gastric cancer (GC) is a common gastrointestinal malignancy worldwide, with the leading incidence and death rates [1]. In 2020, it ranked fifth with over 1 million new cases worldwide and fourth with approximately 769,000 deaths (equivalent to 1 in every 13 tumor-related deaths) [2]. GC presents as a highly molecularly heterogeneous disease that progresses rapidly and, therefore, has a poor prognosis [3]. Chemotherapy combined with targeted therapy significantly prolongs survival and quality of life in patients with GC progression, e.g., paclitaxel (Tax) and platinum-based agents [4]. However, the emergence of acquired drug resistance hinders the effectiveness of chemotherapy and predisposes the patients to treatment failure. Therefore, a deeper understanding of the mechanisms of chemotherapy resistance may provide new ideas and approaches for GC treatment.

Paclitaxel (Tax) is a natural botanical antitumor agent derived from Taxus brevifolia [5]. It functions as a microtubule inhibitor, which induces apoptosis by inhibiting the movement of spindle microtubules and thus causing the cellular G2/M phase block [6]. Due to its unique pharmacological mechanism, paclitaxel is widely used in the clinical treatment of GC, lung cancer and other malignant tumors in the middle and late stages [7,8,9,10]. However, the appearance and development of drug resistance has largely restricted the therapeutic effect of paclitaxel. Similarly, the molecular mechanism of resistance to Tax in GC has not been elucidated.

Covalently closed circular RNA (circRNA) does not possess a linear RNA cap as well as a poly(A) tail structure and presents in the cell as a stable form of closed RNA molecule [11,12]. An increasing number of reports have shown that dysregulated circRNAs conferred oncogenic function in GC and were closely related to tumor chemoresistance [13]. For example, circRNA PVT1 contributed to Tax resistance in GC cells through the regulation of ZEB1 expression by acting as a sponge of miR-124-3p [14]. Nevertheless, the exact role of circRNAs in GC Tax resistance remains questionable. In the present study, we confirmed that circ_002136 was clearly upregulated in GC tissues, and its level correlated with the sensitivity of GC patients to Tax. Specifically, Tax-resistant GC patients had higher circ_002136 expression. Further studies evidenced that circ_002136 could actively sponge miR-16-5p to modulate HMGA1 levels, which in turn induced a Tax-resistant phenotype in GC cells. In the former report, it was presented that the interaction of circ_002136 with FUS, miR-138-5p, SOX13, and SPON2 could retard the growth of glioma [15]. Therefore, the aim of this study was to clarify the molecular mechanisms of circ_002136 in Tax-resistant GC. We hypothesized that circ_002136 interference could sensitize Tax-resistant GC cells to Tax through the miR-16-5p/HMGA1 axis and inhibit the growth and metastasis of Tax-resistant tumor cells, which may provide a new avenue for the treatment of patients with Tax-resistant GC.

## 2 Materials and methods

### 2.1 Patient and sample collection

Tumor tissue specimens (including 20 Tax-sensitive and 17 Tax-resistant tissues) and matched histologically normal tissue specimens located ≥5 cm from the margin of cancer were collected from 37 GC patients who underwent gastrectomy at our institution. The recruited patients signed an informed consent form before surgery. Fresh tissue specimens were harvested under aseptic conditions, rapidly frozen in liquid nitrogen, and stored in a −80°C refrigerator. Inclusion criteria included (1) individuals, aged 18–65 years, who were clinically confirmed diagnosed as GC by pathological examination; (2) individuals who did not administer any preoperative radiotherapy or chemotherapy; and (3) individuals with comprehensive clinicopathological data (including age, gender, degree of tumor differentiation, TNM stating, and lymph node staging).


**Ethics approval and consent to participate:** This study was performed according to institutional guidelines and was approved by Ningbo Mingzhou Hospital Ethics Committee, which consistent with the Declaration of Helsinki.

### 2.2 Cell culture and construction of Tax-resistant cell lines

Two human GC cell lines (SGC7901 and BGC823) were acquired from the Cell Bank of the Chinese Academy of Sciences (Shanghai, China). The corresponding Tax-resistant GC cells (SGC7901/Tax and BGC823/Tax) were created by exposing parental cells to incremental amounts of Tax (Solarbio, China). All cells were grown in RPMI-1640 medium (Gibco, USA) containing 10% fetal bovine serum (Gibco) and 1% penicillin/streptomycin (Gibco). To maintain the resistant phenotype of SGC7901/Tax and BGC823/Tax cells, 5 nM Tax was supplemented to RPMI-1640 medium. All cells were maintained in an atmosphere of 5% CO_2_ at 37°C.

### 2.3 Cell transfection

Cell transfection was achieved in SGC7901/Tax and BGC823/Tax cells by performing Lipofectamine 2000 (Invitrogen, USA). The small-hairpin RNA targeting circ_002136 (si-circ_002136) and its negative control (si-NC), miR-16-5p mimics/inhibitors and its negative control miR-NC were provided by GenePharma (Shanghai, China). To construct stable circ_002136 low-expressing cell lines, lentivirus-mediated short-hairpin RNA against circ_002136 (sh-circ_002136) and its negative control sh-NC were transfected into SGC7901/Tax and BGC823/Tax cells, and 2 mg/mL puromycin was administrated to select stable expression colonies. pcDNA-HMGA1, an overexpression plasmid for HMGA1, was provided by GenePharma, and the empty pcDNA vector was set as negative control (pcDNA). Referring to the manufacturer’s protocols, cells were seeded on six-well plates, 24 h before transfection. After 48 h transfection at 37°C and 5% CO_2_, the cells were harvested for the next experiments.

### 2.4 Ribonuclease R (RNase R) treatment

To check the loop structure of circ_002136, 2 μg of RNA was extracted from GC cells treated with RNase R (Epicenter Technologies). For the experimental group, 1 μg of RNA was mixed with 2 μL of RNase R reaction buffer and 5 U/g of RNase R. For the Mock group, an equal volume of RNase-free water was substituted for RNase R. The prepared reaction system was maintained for additional 15 min at 37°C. Then, TRIzol reagent was immediately added for RNA extraction, followed by the quantitative real-time PCR (qRT-PCR) analysis.

### 2.5 Actinomycin D assay

SGC7901 and BGC823 cells were inoculated on six-well plates (5 × 10^5^ cells/well). To inhibit cell transcription and degrade linear mRNA, cells were then exposed to actinomycin D (2 μg/mL, Sigma) for 0, 4, 8, 12, and 24 h. After actinomycin D treatment, RNA was collected from the cells and the abundance of circ_002136 was presented using qRT-PCR.

### 2.6 circ_002136 subcellular localization prediction

The nuclear and cytoplasmic fractions of SGC7901/Tax and BGC823/Tax cells were departed using the Cytoplasmic & Nuclear RNA Purification Kit (PARIS Kit; Invitrogen). Nucleoplasmic distribution and expression levels of circ_002136 in Tax-resistant GC cells were detected by qRT-PCR. U6 and GAPDH were set as controls for nuclear and cytoplasmic transcripts.

### 2.7 Cellular drug sensitivity assay

Cell viability and cellular Tax sensitivity were assayed by Cell Counting Kit-8 (CCK-8). The transfected cells in each group were resuspended and inoculated into 96-well plates (1 × 10^4^ cells/well) and cultured separately in RPMI-1640 medium for 24 h. Cells were then exposed to several doses of Tax for 72 h. Hereafter, 10 μL of CCK-8 reagent was replenished to each well and maintained for another 2 h at 37°C. Finally, the optical density (OD) values were quantified using an enzyme marker (BioTek Instruments) at 450 nm. The half-maximal inhibitory concentration (IC_50_) values of Tax were determined based on the relationship between the percentage of viable cells and the concentration of Tax.

### 2.8 Wound-healing assays

The proliferative wound-healing assay was carried out to evidence the migratory properties of SGC7901/Tax and BGC823/Tax cells. Briefly, si-NC- or si-circ_002136-transfected cells were seeded into six-well plates for stable growth, followed by scratching when the cells reached 90–95% confluence. In monolayers of cells, pipette tips (200 μL, labeled 0 h) were introduced to create streaks followed by rinsing cells with phosphate buffered saline (PBS) and infusing fresh medium. After continuing incubation for 24 h, the rate of wound area closure was analyzed by light microscopy (Nikon, Tokyo, Japan, magnification ×40) and ImageJ (NIH).

### 2.9 Transwell assay

The abilities of cells invasion were assayed by the Transwell analysis. For the invasion analysis, trypsin (HyClone)-digested 2 × 10^4^ GC cells were first resuspended in 200 μL of serum-free medium and then seeded in Matrigel (BD Biosciences)-coated upper chamber of the Transwell. Five hundred microliters of suitable medium was infused into the bottom chamber. Twenty-four hours later, cells were fixed with paraformaldehyde for 15 min, stained with 0.1% crystalline violet for 10 min, and washed twice with PBS. Five random fields were selected for observation and counting under a microscope (Olympus).

### 2.10 Cell apoptosis analysis

Flow cytometry was used for apoptosis analysis. SGC7901/Tax and BGC823/Tax cells were exposed to specific concentration of si-NC or si-circ_002136 followed by digesting in trypsin for 24 h. Then, cells were collected (1 × 10^5^ cells) and resuspended in a volume of 500 μl of binding buffer. The target cells were then mixed and stained with 5 μL Annexin V/PE and 7-AAD, respectively, for 15 min at room temperature without light, according to the Annexin V PE/7-AAD Apoptosis Detection Kit (KeyGEN Biotech, China). FACScan^®^ (BD Biosciences) was carried out to analyze apoptotic cell ratios.

### 2.11 Dual luciferase reporter gene assay

CircBank (http://www.circbank.cn/) and circinteractome (https://circinteractome.nia.nih.gov/) databases were conducted to predict the targeting relationship between circ_002136 and miR-16-5p, and the target gene of miR-16-5p was demonstrated by TargetScan (http://www.targetscan.org/). The circ_002136 and HMGA1 sequence fragments containing the miR-16-5p complementary sites were amplified and implanted into the reporter vector (pmirGLO; Promega, USA) to produce wild-type luciferase reporter vectors (named as WT-circ_002136 and WT-HMGA1), and the corresponding mutants were constructed (named as MUT-circ_ 002136 and MUT-HMGA1). The above luciferase reporter gene vectors and miR-16-5p mimics or miR-NC were cotransfected into SGC7901/Tax and BGC823/Tax cells to test the miR-16-5p-binding ability. Luciferase activity was obtained and displayed by a dual luciferase reporter gene assay system (Promega), following 48 h transfection.

### 2.12 RNA pull-down assay

The 3′ ends of miR-16-5p and miR-NC were biotin-labeled as Biotin-miR-16-5p and Biotin-NC (Genepharm, China). The biotin-labeled RNA probes were mixed and maintained with streptavidin beads (Thermo Scientific) at 37°C for 2 h. SGC7901/Tax and BGC823/Tax cell lysates were incubated with the probe-coated magnetic beads overnight at 4°C and then eluted with biotin elution buffer. Finally, the biotin-coupled RNA complexes were pulled down and the bound RNA was quantified by qRT-PCR.

### 2.13 qRT-PCR

First, the total RNA was isolated from GC tissues and cells with the help of TRIzol (Beyotime, China) kit. Then, the RNA was reverse transcribed to cDNA in line with the instructions of the reverse transcription kit (PrimeScript™ RT kit, Takara). The cDNA served as template was gently mixed with BeyoFast™ SYBR Green qPCR Mix (Takara) and specific primers. The expression levels of target mRNA and miRNA were amplified and manipulated in accordance with ABI 7500 PCR System (Applied Biosystems). U6 was acted as a reference for miR-16-5p, and GAPDH was functioned as a reference for circ_002136 and HMGA1, respectively. Relative gene expression levels were calculated and normalized by the 2^−∆∆Ct^ method. The primer sequences used were:

circ_002136, F: 5′-CTTTCCGAGACATTTGCTGG-3′,

R: 5′-CATGGAGATCACAATAAGGAACTC-3′;

miR-16-5p, F: 5′-GTCGTGGAGTCGGCAATT-3′,

R: 5′-AUUUGCCAGG UCGGA AUG-3′;

HMGA1, F: 5′-GAAGTGCCAACACCTAAGAGAGACC-3′,

R: 5′-GGTTTCCTTCCTGGAGTTGTGG-3′;

GAPDH, F: 5′-AATGGATTTGGACGCATTGGT-3′,

R: 5′-TTTGCACTGGTACGTGTGTTGAT-3′;

U6, F: 5′-CTGGTAGGGTGCTCGCTTCGGCAG-3′,

R: 5′-CAACTGGTGTCGTGGAGTCGGC-3′.

### 2.14 Western blot analysis

Total proteins were separated from cells of each group in the presence of RIPA lysis buffer (Beyotime) containing protease inhibitors and phosphatase inhibitors. Protein concentrations were confirmed by operating the BCA Protein Assay Kit (Pierce, USA). Thirty micrograms of denatured protein samples were separated by 10% sodium dodecyl sulfate polyacrylamide gel electrophoresis and then transferred to polyvinylidene fluoride membranes. Membranes were blocked in 5% skimmed milk for 2 h and followed by incubation with primary antibody against HMAG1 (1:1,000; Cell Signaling Technologies, USA) at 4°C overnight. Then, all membranes were overlaid and probed with the secondary antibody for an additional 1 h at room temperature. Protein blot images were visualized using an ECL kit (Millipore, USA). GAPDH was treated as an internal reference.

### 2.15 Animal research

For *in vivo* tumorigenesis experiments, BALB/c nude mice (male, 4–6 weeks old) were obtained from Shanghai Animal Experiment Center (Shanghai, China) and randomly assigned to four groups: sh-NC + PBS, sh-circ_002136 + PBS, sh-NC + Tax, and sh-circ_002136 + Tax. circ_002136 (sh-circ_002136) of shRNA lentiviral vector and its negative control (sh-NC) were provided by GenePharma. Orthotopic transplantation models were constructed by subcutaneously injecting 4 × 10^6^ SGC7901/Tax cells stably transfected with sh-circ_002136 or sh-NC into the right side of nude mice. After 7 days, the sh-NC + Tax and sh-circ_002136 + Tax groups were injected intraperitoneally with 30 mg/kg Tax every 3 days, while the sh-NC + PBS and sh-circ_002136 + PBS groups were injected with equal volume of PBS. Tumor size was recorded and volume was calculated according to the formula = 0.5 × length × width^2^. Nude mice were executed after 30 days, and the tumors were removed and weighed immediately. The expression of circ_002136 and miR-16-5p was analyzed by qRT-PCR. The surgically excised tumor tissues were paraffin-embedded for tissue sectioning (6 μm) and subjected to immunohistochemistry (IHC) [16]. Tissue sections were incubated with anti-HMGA1 (1:500) for 60 min. Biotinylated goat anti-rabbit serum IgG (Beyotime Institute of Biotechnology) was used as a secondary antibody. The antigen/antibody complexes were visualized with diaminobenzidine (Dako, Carpinteria, CA, USA) and re-stained with hematoxylin. Images were analyzed using an Institute of Medical Statistics imaging processing system.

### 2.16 Statistical analysis

The experiments used were performed three times independently and the data obtained are expressed as mean ± standard deviation. Statistical differences between the two or multiple groups were determined using Student’s *t*-test or one-way ANOVA. Statistical analysis was performed in SPSS 22.0 statistical software (Chicago, IL, USA) and graphs were plotted in GraphPad Prism 7.0 software. A *P*-value of less than 0.05 was considered as the criterion for statistical significance.

## 3 Results

### 3.1 hsa_circ_002136 expression is upregulated in Tax-resistant GC tissues and cells

**Figure 1:**
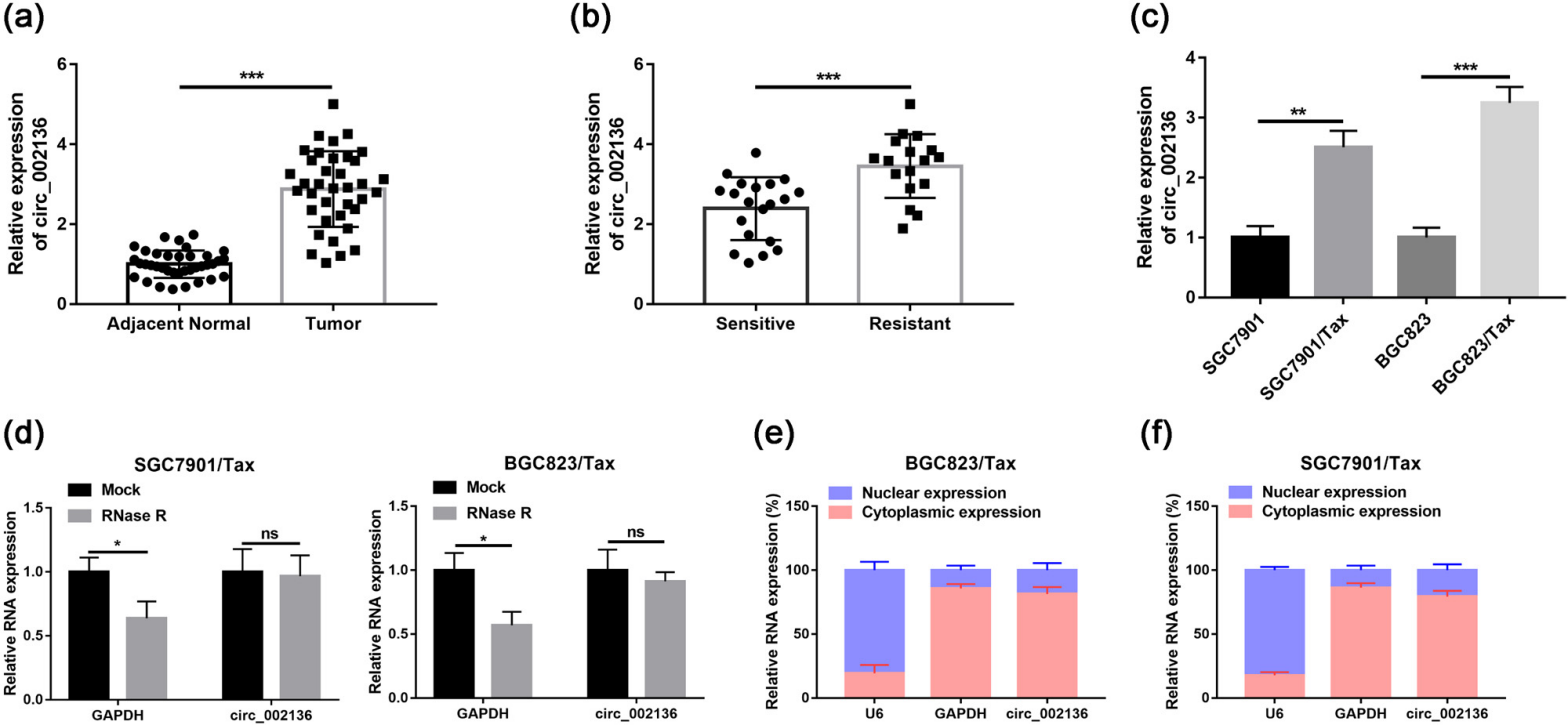
circ_002136 was overexpressed in the GC tissues and cell lines. (a) circ_002136 expression was measured in adjacent normal tissues and tumor tissues by qRT-PCR. (b) The expression of circ_002136 was determined in patients responding or not responding to paclitaxel treatment. (c) Relative expression of circ_002136 was detected by qRT-PCR in SGC7901, SGC7901/Tax, BGC823, and BGC823/Tax cells. (d) The relative levels of circ_002136 and GAPDH mRNA were examined by qRT-PCR after treatment with RNase R in SGC7901/Tax and BGC823/Tax cells. (e and f) The qRT-PCR test was applied to determine the subcellular location of circ_00081001 in SGC7901/Tax and BGC823/Tax cells. *P < 0.05, **P < 0.01, and ***P < 0.001.

The expression of circRNAs is closely related to the gene regulation of GC. Certainly, the properties of circ_002136 also need to be studied. A search in circBase and circBank revealed that hsa_circ_002136 (hsa_circ_0000005, hsa_circCDK11A_001) was originated from the linear *CDK11A* gene with the position of chr1: 1,586,822–1,650,894, which was formed by the transcript *CDK11A-VT1* (cyclin-dependent kinase 11A transcript variant 1) closed into a loop with a length of 49,639 bp. Thereafter, to investigate the role of circ_002136 in regulating drug resistance progression in GC, we first determined the circ_002136 expression characteristics by qRT-PCR in 37 pairs of GC tissues. The obtained data showed that circ_002136 was significantly upregulated in GC tissues compared to paired non-tumor tissues (Figure 1a). Moreover, we also examined the expression levels of circ_002136 in Tax-sensitive (*n* = 20) and Tax-resistant (*n* = 17) GC tissues. The experimental results confirmed that circ_002136 expression was significantly enriched in Tax-resistant tissues compared to Tax-sensitive tissues (Figure 1b). In addition, the matched Tax-resistant GC cells (SGC7901/Tax and BGC823/Tax) showed higher expression of circ_002136 compared to parental SGC7901 and BGC823 cells (Figure 1c). Next, we analyzed the stability and localization of circ_002136 in Tax-resistant GC cells. The total RNA from GC cell lines was treated with or without RNase R. It was revealed that circ_002136 was resistant to nucleic acid exonuclease-mediated RNA degradation (expression was only slightly decreased compared to GAPDH mRNA), indicating that circ_002136 was more stable (Figure 1d). The function of circRNA is largely influenced by subcellular localization. Therefore, we analyzed cytoplasmic and nuclear RNA from GC cells by qRT-PCR to determine the subcellular localization of circ_002136. Figure 1e and f shows that circ_002136 had a ring-like structure and is mainly distributed in the cytoplasm. The above results suggest that circ_002136 may become a novel biomarker for GC.

### 3.2 Silencing of hsa_circ_002136 increases Tax sensitivity of GC cells

**Figure 2:**
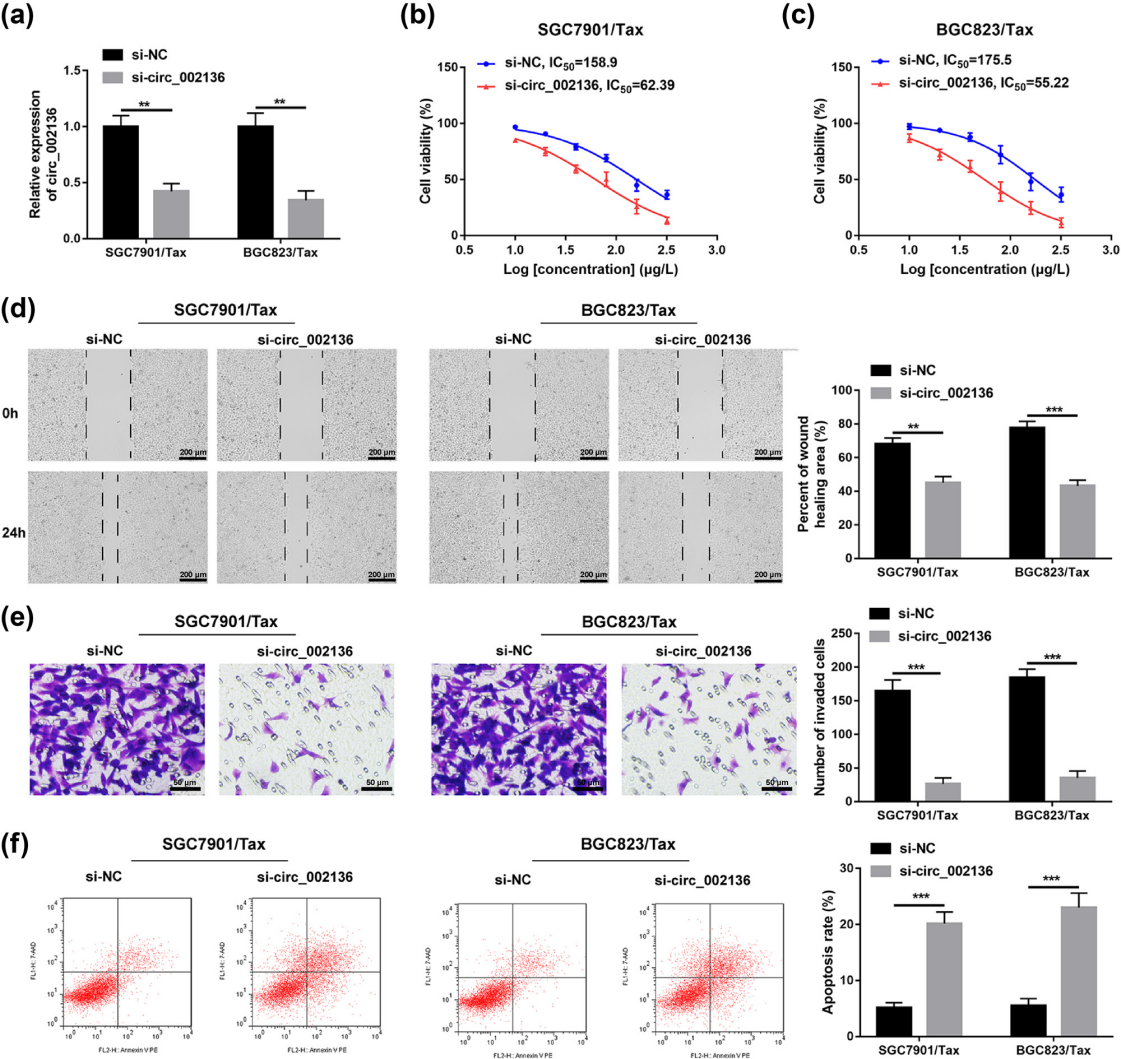
Downregulation of circ_002136 inhibited paclitaxel resistance, migration, invasion and induced apoptosis in GC. (a) circ_002136 expression in SGC7901/Tax and BGC823/Tax cells with the transfection of si-circ_002136 or si-NC. (b and c) Cell viability and IC_50_ of paclitaxel in cells with the transfection of si-circ_002136 or si-NC after exposure to various doses of paclitaxel for 72 h. (d and e) Cell migration and invasion in cells with the transfection of si-circ_002136 or si-NC. Scale bars = 200, 50 μm. (f) Cell apoptosis in cells with the transfection of si-circ_002136 or si-NC. **P < 0.01, ***P < 0.001.

Afterwards, to further investigate the effect of circ_002136 on Tax-sensitive GC cells and Tax-resistant GC cells, small-interfering RNA (si-RNA) against circ_002136 was constructed and determined the si-circ_002136 knockdown efficiency in SGC7901/Tax and BGC823/Tax cell lines. qRT-PCR data indicated that si-circ_002136 transfection resulted in significantly lower circ_002136 levels in SGC7901/Tax and BGC823/Tax cells, compared to the corresponding controls (Figure 2a). As shown in Figure 2b and c, compared to Tax-resistant GC cells exposed to si-NC, the decrease of IC_50_ values was presented in si-circ_002136-transfected SGC7901/Tax and BGC823/Tax cells, indicating that both cells increased sensitivity to Tax. As expected, the migration and invasion abilities of SGC7901/Tax and BGC823/Tax cells were also weakened by the intervention of si-circ_002136 (Figure 2d and e). Besides, the flow cytometry analysis also revealed that silencing of circ_002136 activated the apoptosis of Tax-resistant GC cells (Figure 2f). In conclusion, the above experimental results suggest that circ_002136 inhibition enhances Tax-induced GC cytotoxicity and hinders GC progression *in vitro*.

### 3.3 hsa_circ_002136 knockdown enhances the Tax sensitivity of Tax-resistant GC cells by upregulating miR-16-5p

**Figure 3:**
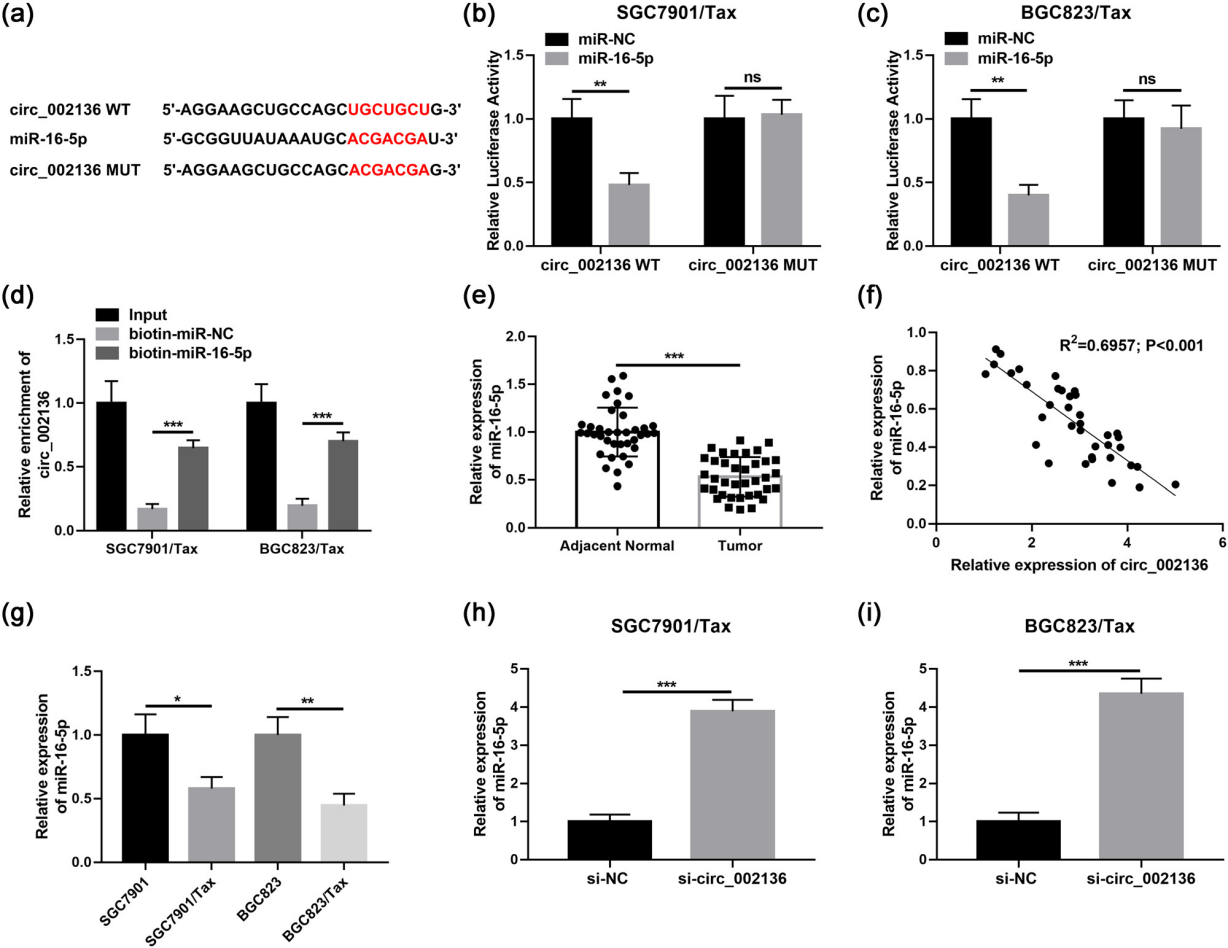
miR-16-5p is a binding target of circ_002136. (a) The complementary sequence between circ_002136 and miR-16-5p. (b and c) Luciferase activity was detected in cells with the co-transfection of circ_002136-WT or circ_002136-MUT and miR-16-5p mimic or miR-NC. (d) circ_002136 expression in cells with the transfection of biotin-miR-NC or biotin-miR-16-5p after RNA pull-down. (e) miR-16-5p expression in adjacent normal tissues and GC tissues. (f) circ_002136 and miR-16-5p expression association in GC tissues. (g) miR-16-5p expression in SGC7901, SGC7901/Tax, BGC823, and BGC823/Tax cells. (h and i) miR-16-5p expression in cells after circ_002136 downregulation. *P < 0.05, **P < 0.01, ***P < 0.001.

**Figure 4:**
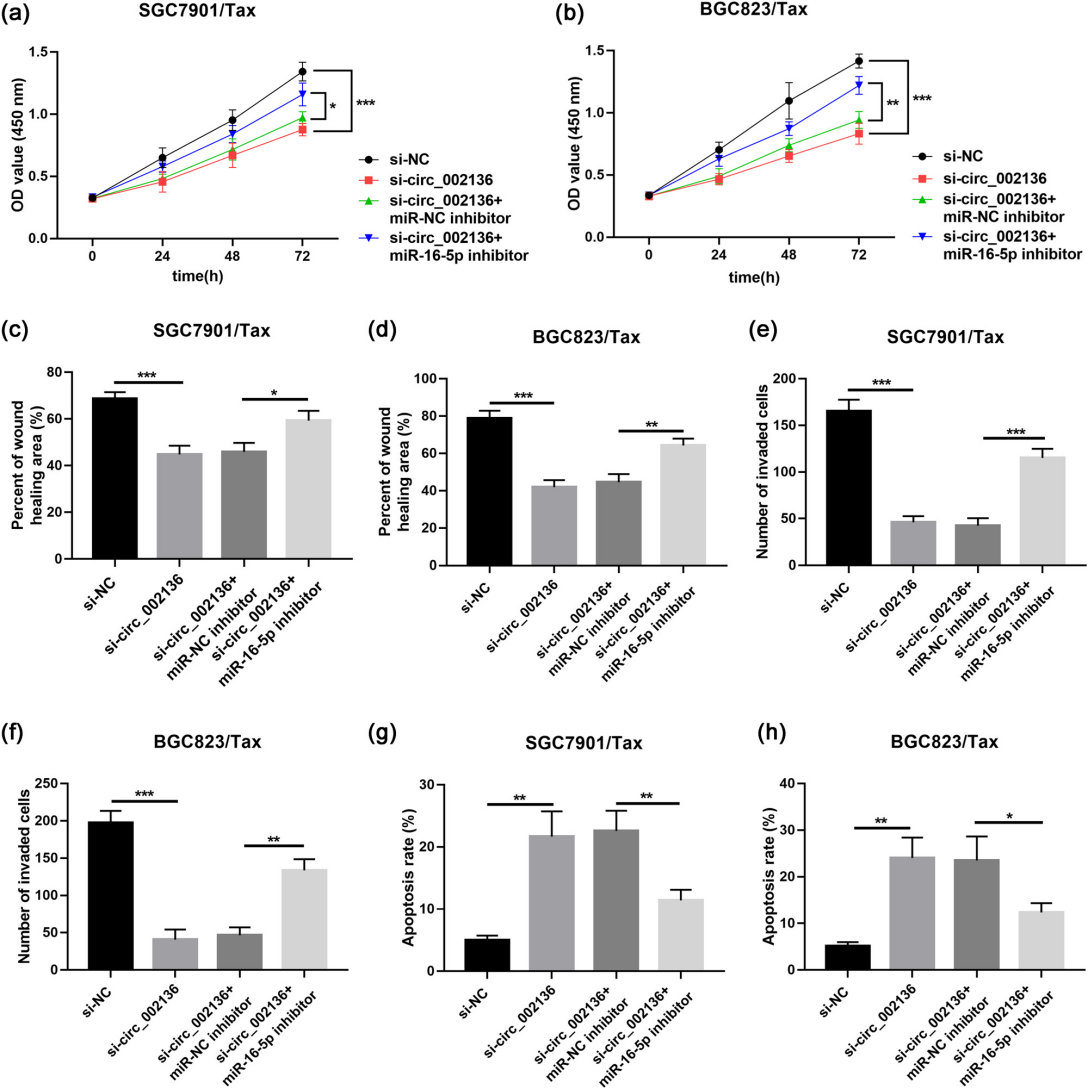
Inhibition of miR-16-5p reversed the effects of circ_002136 knockdown in GC. (a and b) Cell viability after paclitaxel treatment in SGC7901/Tax and BGC823/Tax cells. (c and d) Migration, (e and f) invasion, and (g and h) apoptosis in cells with the transfection of si-NC, si-circ_002136, si-circ_002136 + miR-NC inhibitor, or miR-16-5p inhibitor. *P < 0.05, **P < 0.01, ***P < 0.001.

The regulation mechanism of circRNA on miRNA activity and its abundant or tissue-specific expression patterns make circRNA a potential molecular marker for diagnosing disease. To explore the molecular mechanisms of circ_002136 in GC, we used circBank (http://www.circbank.cn/) and circinteractome (https://circinteractome.nia.nih.gov/) bioinformatics prediction software to find potential target miRNAs for circ_002136. Finally, miR-16-5p was identified as a direct target (Figure 3a). Figure 3a shows the binding sites of circ_002136 to miR-16-5p. To verify the direct interaction between the above two RNAs, we constructed luciferase reporter genes (wild-type circ_002136 and mutant circ_002136) and cotransfected them with miR-16-5p mimic or miR-NC into SGC7901/Tax and BGC823/Tax cells. The obtained results demonstrated that miR-16-5p mimics transfection effectively attenuated the luciferase activity of the circ_002136-WT reporter vector in SGC7901/Tax and BGC823/Tax cells with significant reductions of 52 and 60% but had no effect on the luciferase activity of circ_002136-MUT (Figure 3b and c). In addition to analyzing the luciferase activity, we performed RNA pull-down analysis on specific biotin-labeled miR-16-5p by using SGC7901/Tax and BGC823/Tax cell lysates. As shown in Figure 3d, the biotin-miR-16-5p was specifically enriched for circ_002136. Meanwhile, we determined miR-16-5p expression in 37 GC patient tissues using qRT-PCR. The miR-16-5p levels in GC tissues (both Tax-sensitive GC tissues and Tax-resistant GC tissues) compared to non-tumor tissues were lower (Figure 3e). Correlation analysis showed that miR-16-5p was negatively correlated with circ_002136 in GC tissues (Figure 3f). Furthermore, miR-16-5p expression was dwindled in SGC7901/Tax and BGC823/Tax cells compared to parental cells SGC7901 and BGC823 (Figure 3g). Finally, we transfected circ_002136 siRNA into GC cells to assess miR-16-5p expression. The results indicated that knockdown of circ_002136 apparently enhanced the level of miR-16-5p in SGC7901/Tax and BGC823/Tax cells (Figure 3h and i). Our experimental results suggest that circ_002136 may mediate GC cell Tax resistance via miR-16-5p. Next, to elucidate whether miR-16-5p plays a role in circ_002136-mediated Tax resistance in GC cells, we co-transfected si-circ_002136 and miR-16-5p into SGC7901/Tax or BGC823/Tax cells for rescue experiments. The present results of CCK-8 assay validated that miR-16-5p inhibitor partially attenuated the inhibitory effect of circ_002136 silencing on Tax resistance in SGC7901/Tax and BGC823/Tax cells (Figure 4a and b). Similarly, miR-16-5p inhibitor also partially reversed the hampering effect of knockdown of circ_002136 on the migration and invasion of SGC7901/Tax and BGC823/Tax cells, as well as the promotion of apoptosis in SGC7901/Tax and BGC823/Tax cells (Figure 4c–h). All data suggest that circ_002136 can directly target miR-16-5p and regulate Tax resistance in GC cells.

### 3.4 HMGA1 is a new target of miR-16-5p in Tax-resistant GC cells

**Figure 5:**
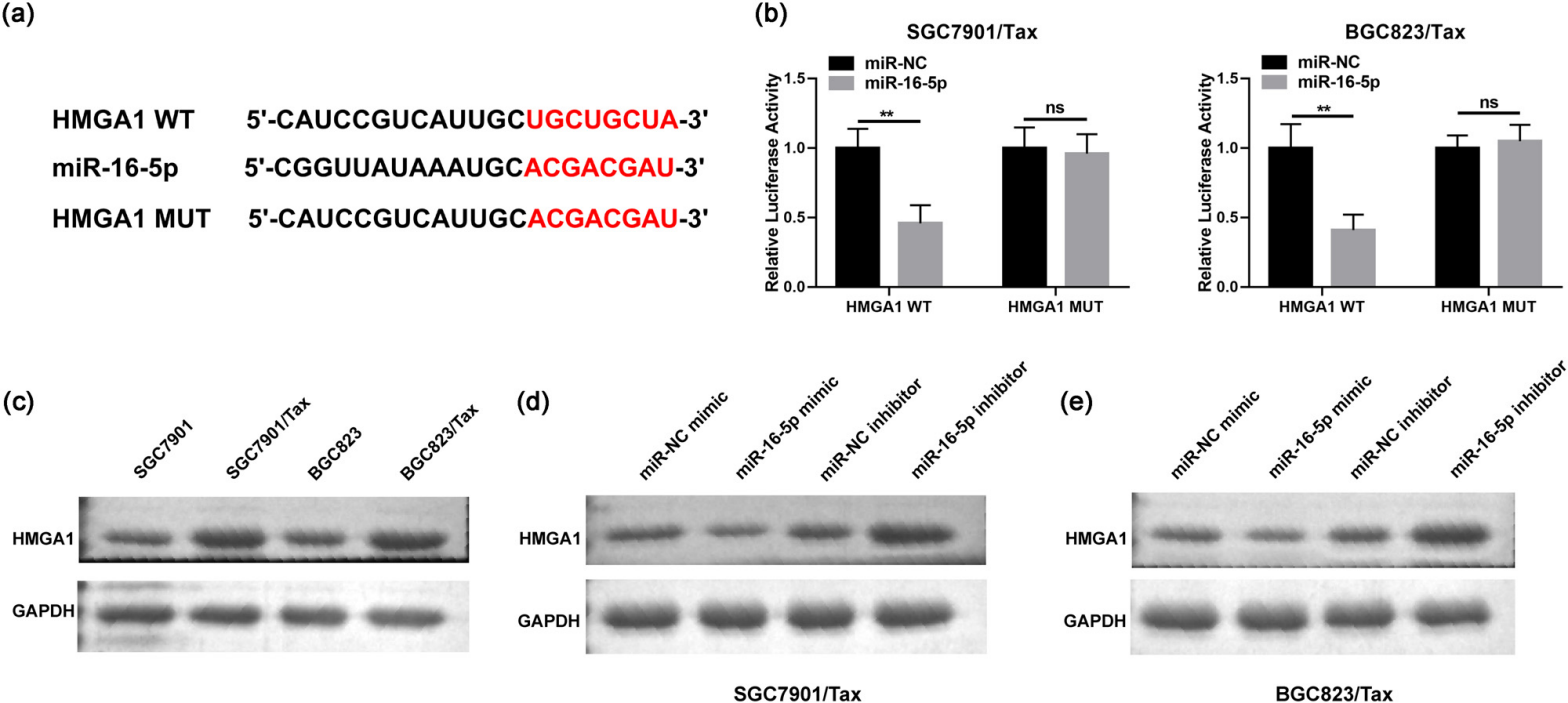
The relationship of miR-16-5p and HMGA1. (a) The complementary sequence between miR-16-5p and HMGA1. (b) Luciferase activity was examined in cells with the co-transfection of HMGA1-WT or HMGA1-MUT and miR-16-5p mimic or miR-NC. (c) HMGA1 expression in SGC7901, SGC7901/Tax, BGC823, and BGC823/Tax cells. (d and e) HMGA1 protein expression level in cells with the transfection of miR-NC, miR-16-5p mimic, miR-NC inhibitor or miR-16-5p inhibitor. *P < 0.05, **P < 0.01, ***P < 0.001.

circRNAs/miRNAs/mRNAs crosstalk has a potential role as a tumor suppressor in GC cell lines [17]. To further investigate the mechanism of ceRNA crosstalk in GC, the bioinformatics online software TargetScan (http://www.targetscan.org/vert_72/) was applied to predict the potential targets of miR-16-5p. As shown in Figure 5a, as predicted, the HMGA1 3′-UTR had binding sites to miR-16-5p. The luciferase fluorescence activity measurements showed that miR-16-5p mimic caused sharply diminished the luciferase activity signal in WT-HMGA1-transfected SGC7901/Tax and BGC823/Tax cells, whereas MUT-HMGA1 had no such effect (Figure 5b). In addition, HMGA1 protein appeared high abundance expression in both SGC7901/Tax and BGC823/Tax cells, with an expression trend opposite to miR-16-5p (Figure 5c). To further verify the effect of miR-16-5p on HMGA1 expression, we examined the levels of HMGA1 protein after stimulation with miR-16-5p mimics and inhibitors. As shown in Figure 5d and e, weakened miR-16-5p level resulted in elevated HMGA1 level in SGC7901/Tax and BGC823/Tax cells. In contrast, overexpression of miR-16-5p caused attenuated expression of HMGA1 protein. These results suggest that HMGA1 is a direct target of miR-16-5p.

### 3.5 Restored expression of miR-16-5p suppresses Tax resistance, cell migration, invasion, and apoptosis in Tax-resistant GC cells through downregulation of HMGA1

**Figure 6:**
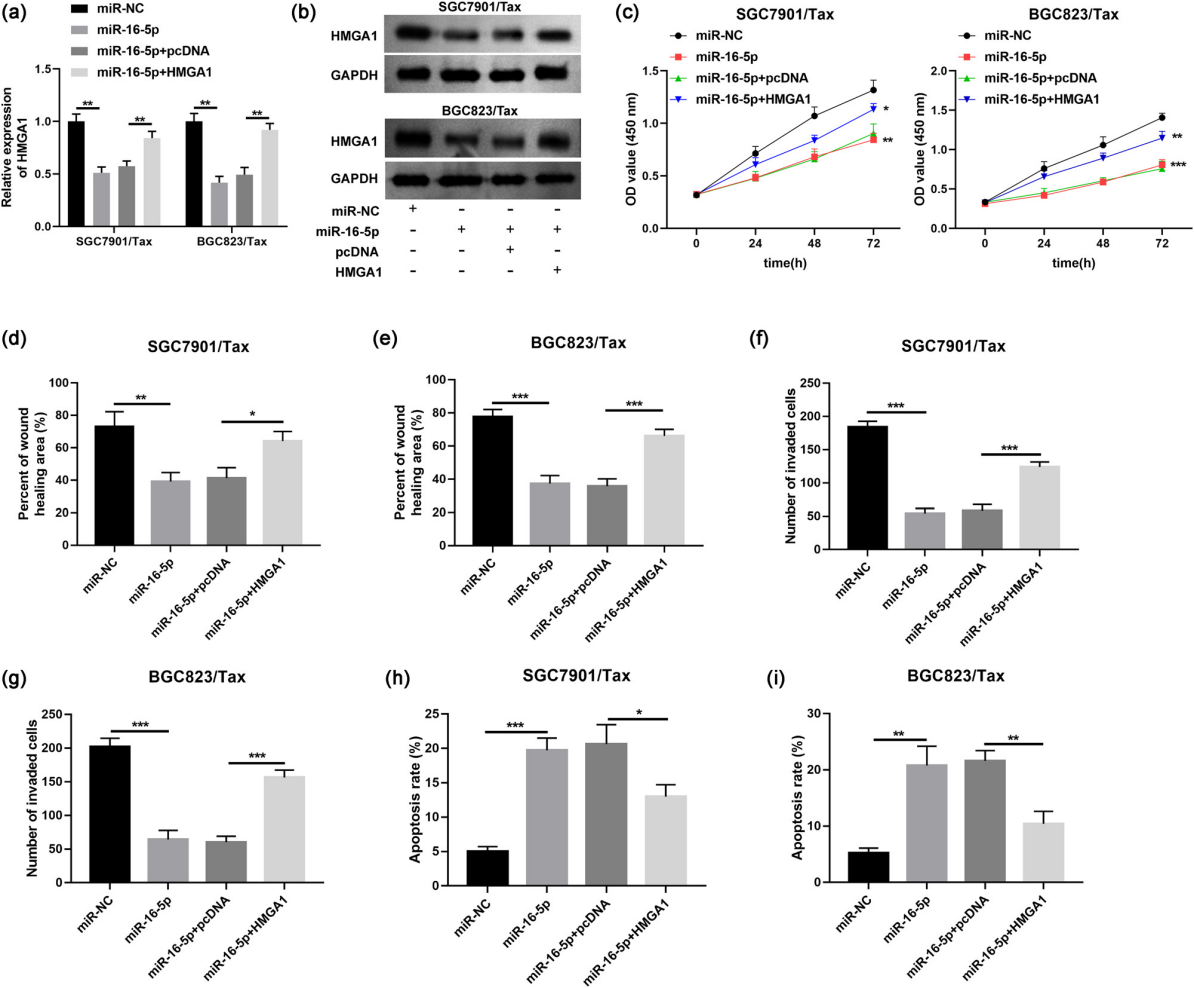
miR-16-5p affected paclitaxel resistance, migration, invasion, and apoptosis by regulating HMGA1. (a) HMGA1 mRNA and (b) protein levels in SGC7901/Tax and BGC823/Tax cells with the transfection of miR-NC, miR-16-5p mimic, miR-16-5p mimic + pcDNA, or pcDNA HMGA1. (c) Cell viability after paclitaxel treatment in SGC7901/Tax and BGC823/Tax cells. (d and e) Migration, (f and g) invasion, and (h and i) apoptosis in cells with the transfection of miR-NC, miR-16-5p mimic, miR-16-5p mimic + pcDNA, or pcDNA HMGA1. *P < 0.05, **P < 0.01, ***P < 0.001.

Previous studies demonstrated that HMGA1 was an oncogene associated with GC tumors, which was involved in tumor cell migration, invasion, and cisplatin resistance. To explore the role of HMGA1 on Tax resistance in GC, we transfected miR-NC, miR-16-5p mimic, miR-16-5p mimic + pcDNA, or pcDNA HMGA1 into SGC7901/Tax and BGC823/Tax cells. qRT-PCR and western blot analyses confirmed that HMGA1 mRNA and protein levels were downregulated in both SGC7901/Tax and BGC823/Tax cells after the administration of miR-16-5p mimic, but introduction of HMGA1 overexpression plasmid in these cells improved the HMGA1 level after miR-16-5p overexpression (Figure 6a and b). Cells were treated with specific amount of paclitaxel and then subjected to CCK-8 assays. The obtained experimental data indicated that increased HMGA1 level partially restored Tax sensitivity mediated by miR-16-5p overexpression in SGC7901/Tax and BGC823/Tax, increasing cellular Tax resistance, as evidenced by increased OD 450 values (Figure 6c). Subsequent wound-healing, Transwell, and flow cytometry analyses showed that pcDNA HMGA1 effectively weakened the inhibitory effect on SGC7901/Tax and BGC823/Tax cells’ migration (Figure 6d and e), invasion (Figure 6f and g), and promotion of apoptosis (Figure 6h and i) induced by miR-16-5p overexpression. These results confirmed that HMGA1 could indeed affect paclitaxel resistance in GC cells.

### 3.6 Knockdown of hsa_circ_002136 enhances Tax sensitivity of GC *in vivo* and inhibits tumor malignant progression

**Figure 7:**
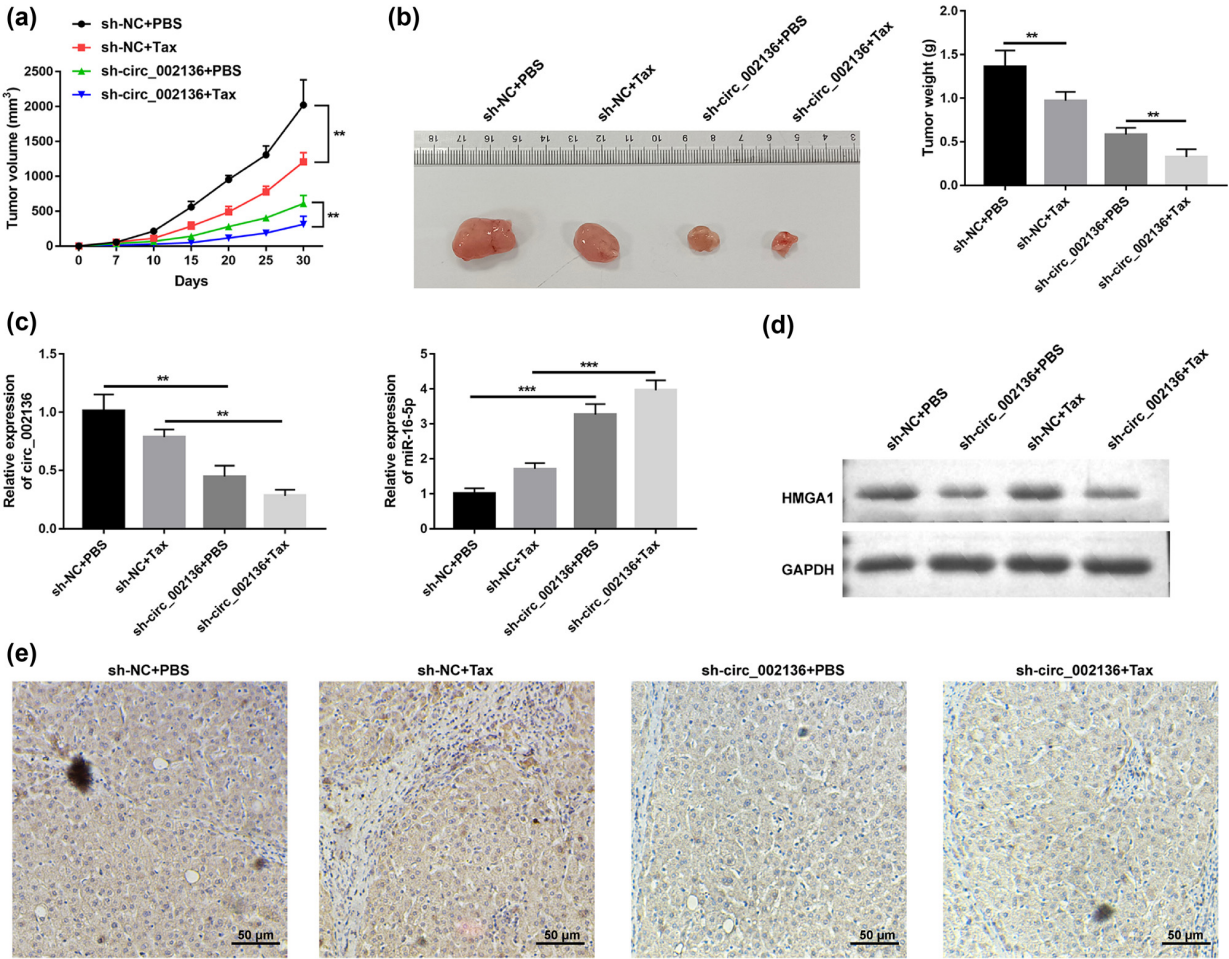
circ_002136 inhibition restrained tumor growth and sensitizes gastric cells to paclitaxel *in vivo*. (a) Growth curve of the tumors. (b) Representative images (right) and mean weight (left) of the xenograft tumors. (c) circ_002136 and miR-16-5p expression levels were examined by qRT-PCR. (d and e) HMGA1 expression level in tumors was detected by western blot (d) and IHC (e). Scale bar = 50 μm. *P < 0.05, **P < 0.01, ***P < 0.001.

To confirm the effect of circ_002136 on the growth of Tax-resistant GC tumors *in vivo*, we observed tumor growth after subcutaneous inoculation of sh-circ_002136 stably transfected SGC7901/Tax cells via xenograft tumor model. As shown in Figure 7a and b, compared with the sh-NC group, the tumor size and tumor weight in the sh-NC + Tax group treated with Tax were downregulated, which suggested that Tax treatment could control GC tumor development *in vivo*. Interestingly, we found that downregulation of circ_002136 enhanced the anti-tumor effect of Tax (Figure 7a and b). Correspondingly, qRT-PCR results showed an obvious decrease in circ_002136 in the sh-circ_002136 groups compared with the sh-NC groups (Figure 7c). Western blot and immunohistochemical analyses also presented that knockdown of circ_002136 reduced HMGA1 expression *in vivo* (Figure 7d and e). It can be concluded that silencing of circ_002136 induced Tax sensitivity of GC *in vivo* via the miR-16-5p/HMGA1 axis.

## 4 Discussion

Chemotherapy can improve the endpoints of progression-free survival and overall remission rate of cancer patients and is an effective treatment for advanced cancer patients who cannot undergo surgery. As a highly effective microtubulin cytotoxin, Tax can “freeze” the mitotic spindle of rapidly dividing cancer cells and induce G2/M cell cycle arrest [18]. Therefore, Tax has significant anti-tumor activity and has gradually developed into a first-line chemotherapeutic agent for the treatment of GC. However, most GC patients have developed drug resistance when treated with Tax. And the resistance mechanism of Tax is still in the exploration stage. It is hypothesized that the mechanism of Tax resistance involves multiple biological processes, including alterations in drug targets, alterations in intercellular signaling, and changes in cell cycle check proteins [19,20]. The stably expressed circRNAs are not only involved in the malignant progression of tumors, but there is now a wealth of data suggesting that circRNAs are also associated with Tax resistance in tumors. Circ-PVT1 could confer paclitaxel resistance to GC cells through upregulation of miR-124-3p-mediated ZEB1 [14]. CircCELSR1 shared the same response element with miR-1252 and bound competitively with miR-1252 to regulate FOXR2 expression, which in turn reduced the sensitivity of ovarian cancer cells to Tax [10]. circ_0006528 was highly expressed in Tax-resistant breast cancer tissues and cells and might regulate Tax resistance by binding to miR-1299 [21]. Therefore, in this study, we identified a novel circRNA associated with Tax resistance, circ_002136, which was significantly enriched in both Tax-sensitive and Tax-resistant GC tissues but presented at higher levels in Tax-resistant tissues than in Tax-sensitive tissues, implying that circ_002136 could play a relatively important role in both Tax-resistant and Tax-sensitive GC. Accordingly, we downregulated circ_002136 in Tax-resistant GC cells (SGC7901/Tax and BGC823/Tax) *in vivo* and *in vitro* to deepen the understanding of the potential mechanisms for chemoresistance in GC.

miR-16-5p has been observed to be involved in the propagation of chemoresistance in tumor cells and to predict cancer progression. Previous studies have found that miR-16-5p served as a potential biomarker, indicating a strong risk factor for GC progression [22]. Furthermore, miR-16-5p overexpression improved TAMR sensitivity in breast cancer cells [23] and cisplatin treatment sensitivity in osteosarcoma cells [24] and participated in anti-adriamycin chemoresistance in cervical cancer through PDK4-mediated metabolic reprogramming [25]. Therefore, this study determined whether miR-16-5p was involved in the induction of paclitaxel-resistant phenotype in GC cells. Here, we found that circ_002136 had endogenous sponge-like effect on miR-16-5p in GC. First, bioinformatics online software and luciferase reporter gene assay proved the targeted binding effect between circ_002136 and miR-16-5p. Second, miR-16-5p was lower expressed in Tax-resistant GC cells and tissues. Finally, circ_002136 silencing-induced Tax sensitivity in GC cells could be overridden by miR-16-5p knockdown. These data imply that circ_002136 may mediate Tax resistance in GC via miR-16-5p, suggesting a potential function of circ_002136/miR-16-5p as a drug-associated gene in cancer therapy.

An interesting finding in our study was that high mobility group A1 (HMGA1) could be involved in the induction of Tax resistance in GC cells. HMGA1 competitively was bound to miR-16-5p in GC cells to reduce its expression. It has been reported that the dysregulation of HMGA1 was correlated with the cancer drug-resistant phenotype [26,27]. Recent studies have shown that HMGA1 bound specifically to miR-218 to mediate cisplatin resistance in GC cells [28]. In our results, miR-16-5p could target HMGA1 3′-UTR, and HMGA1 overexpression promoted the progression of Tax resistance in GC cells. Also, HMGA1 overexpression partially reversed the inhibitory effect of miR-16-5p mimic on cell progression. Finally, knockdown of circ_002136 *in vivo* induced an increase in miR-16-5p and restrained HMGA1 level and ultimately curbed the growth of Tax-resistant tumors. Taken together, these results represent that circ_002136 causes activation of the miR-16-5p/HMGA1 pathway and induce a Tax-resistant phenotype in GC cells. Therefore, targeting circ_002136 may be a new goal to overcome paclitaxel resistance in GC.

## 5 Conclusion

To conclude, this study revealed for the first time that circ_002136 could be activated in Tax-resistant GC tissues and cells, and higher circ_002136 levels were related to tumor malignant phenotypes. In addition, *in vitro* experiments showed that knockdown of circ_002136 increased Tax sensitivity in GC cells, and also *in vivo* tests confirmed that silencing of circ_002136 controlled the growth of Tax-resistant GC tumors. Mechanically, the targeted binding of circ_002136 to miR-16-5p could unlock the translational repression of HMGA1, a downstream target gene of miR-16-5p. In summary, we provided a promising circRNA-targeted therapy for GC patients, in which the circ_002136/miR-16-5p/HMGA1 axis might provide a new idea for cancer treatment.
